# Identification of immunodominant antigens in canine leptospirosis by Multi-Antigen Print ImmunoAssay (MAPIA)

**DOI:** 10.1186/s12917-014-0288-2

**Published:** 2014-12-03

**Authors:** Sabrina Thomé, Carolina Lessa-Aquino, Albert Icksang Ko, Walter Lilenbaum, Marco Alberto Medeiros

**Affiliations:** Laboratory of Veterinary Bacteriology, Department of Microbiology and Parasitology, Universidade Federal Fluminense, Niterói, RJ Brazil; Fiocruz, Bio-Manguinhos, Laboratory of Recombinant Technology, Avenida Brasil, 4365, Manguinhos, 21045-900 Rio de Janeiro, RJ Brasil; School of Public Health, Department of Epidemiology of Microbial Diseases, Yale University, 60 College Street, Downtown, 06510 New Haven, USA

**Keywords:** Leptospirosis, Dogs, MAPIA, LipL32, Lig proteins

## Abstract

**Background:**

The microscopic agglutination test (MAT), the standard method for serological diagnosis of leptospirosis, may present limitations regarding its sensitivity. Current studies suggest that *Leptospira* immunoglobulin-like (Lig) proteins and LipL32 are of particular interest as serodiagnostic markers since they are present only in pathogenic species of the *Leptospira* genus. The purpose of this study was to identify leptospiral immunodominant proteins that are recognized by canine sera from diseased dogs.

**Results:**

A total of 109 dogs were studied, including seroreactive dogs (MAT ≥800) and dogs with no seroreactivity detectable by MAT. Eight recombinant fragments (31–70 kDa) of pathogenic *Leptospira* were tested for their use as diagnostic markers for canine leptospirosis using the Multi-antigen Print Immunoassay (MAPIA) platform: LigB [582-947aa] from *L. interrogans* serovar Pomona, *L. interrogans* serovar Copenhageni and *L. kirschneri* serovar Gryppotyphosa, LigB [131-649aa] from *L. interrogans* serovar Copenhageni, *L. interrogans* serovar Canicola and *L. kirschneri* serovar Gryppotyphosa, LigA [625-1224aa] *L. interrogans* serovar Copenhageni and LipL32 from *L. interrogans* serovar Copenhageni. The data were analyzed and ROC curves were generated. Altogether, LigB [131-649aa] *L. interrogans* Canicola, LigB [131-649aa] *L. kirschneri* Gryppotyphosa and LipL32 *L. interrogans* Copenhageni showed best accuracy (AUC = 0.826 to 0.869), with 70% specificity and sensitivity ranging from 89% to 95%.

**Conclusions:**

These results reinforce their potential as diagnostic candidates for the development of new methods for the serological diagnosis of canine leptospirosis.

## Background

Leptospirosis in dogs may manifest with a wide range of symptoms, from fever to liver and kidney failure, jaundice and bleeding. It is a widespread life-threatening disease with zoonotic potential, particularly in tropical areas [1,2], where characteristics such as climate, topography and also regularity of veterinary assistance affect the prevalence of the disease [3].

The early diagnosis becomes a priority for allowing therapeutic incursions and more effective control measures [4]. However, the most employed serological method for the diagnosis of leptospirosis – the microscopic agglutination test (MAT) - may not present adequate sensitivity, particularly in detecting early disease. Additionally, it requires specific laboratory facilities and is too laborious, offering risk to the operator [5]. The MAT detects agglutinating antibodies and paired serum samples with an interval of at least 10 days are needed to diagnose acute infection [6]. Due to these limitations, recent studies have applied proteomic approaches in order to identify novel protein antigens for the development of alternative diagnostic tests [7]. Currently, a variety of alternative methods based on recombinant proteins are under development, such as ELISAs and immunoblotting assays, and have shown encouraging results [8,5,9,10].

Several reported serodiagnostic assays target the leptospiral immunoglobulin-like (Lig) proteins as antigen [10]. It has been demonstrated that the *lig* genes are highly conserved among different leptospiral species and present exclusively among pathogenic leptospires [11]. Besides, the lig proteins are exposed on the surface of the bacterium and have been identified not only as diagnostic markers during early leptospirosis but also as potential candidates for vaccine development [8,12]. Additionally, LipL32 is also a protein with remarkable importance in this context, since it is the most abundant outer membrane lipoprotein of leptospires and seems to be highly immunogenic [13,14].

The purpose of this study was to identify immunodominant proteins of leptospires that are recognized by sera from healthy or diseased dogs, which can be employed in the development of a novel diagnostic test for canine leptospirosis.

## Results

According to the MAT results, reactions against members of serogroup Icterohaemorrhagiae (serovars Icterohaemorrhagiae and Copenhageni) were the most common, representing 86.7% (26/30) of all seroreactive samples. Seroreactivity against serogroups pomona (10%, 3/30) and grippotyphosa (3.3%, 1/30) were also detected.

**Figure 1:**
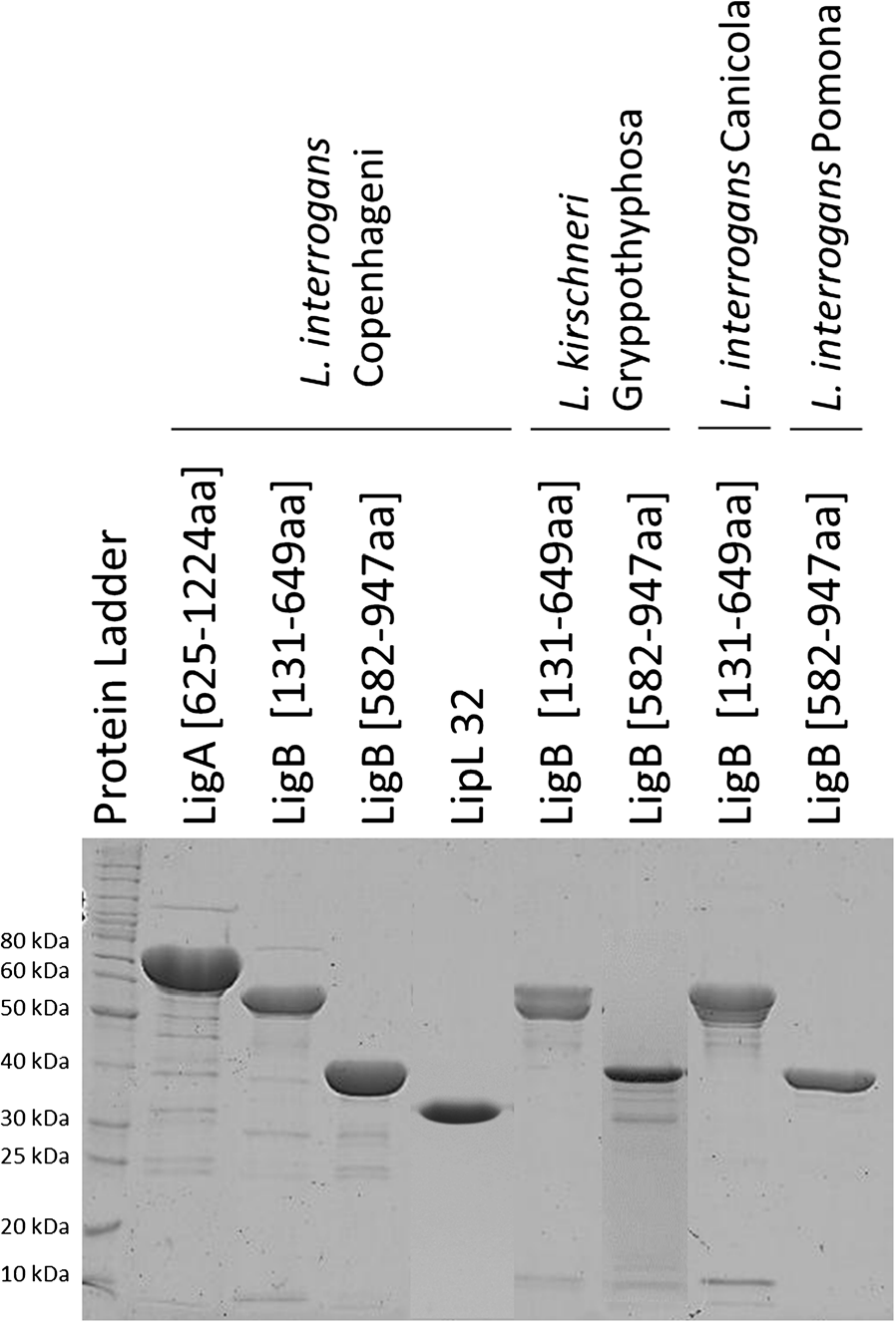
**Homogeneity of the recombinant proteins used in MAPIA.** SDS PAGE 12,5% of each purified recombinant protein used in this study.

**Figure 2:**
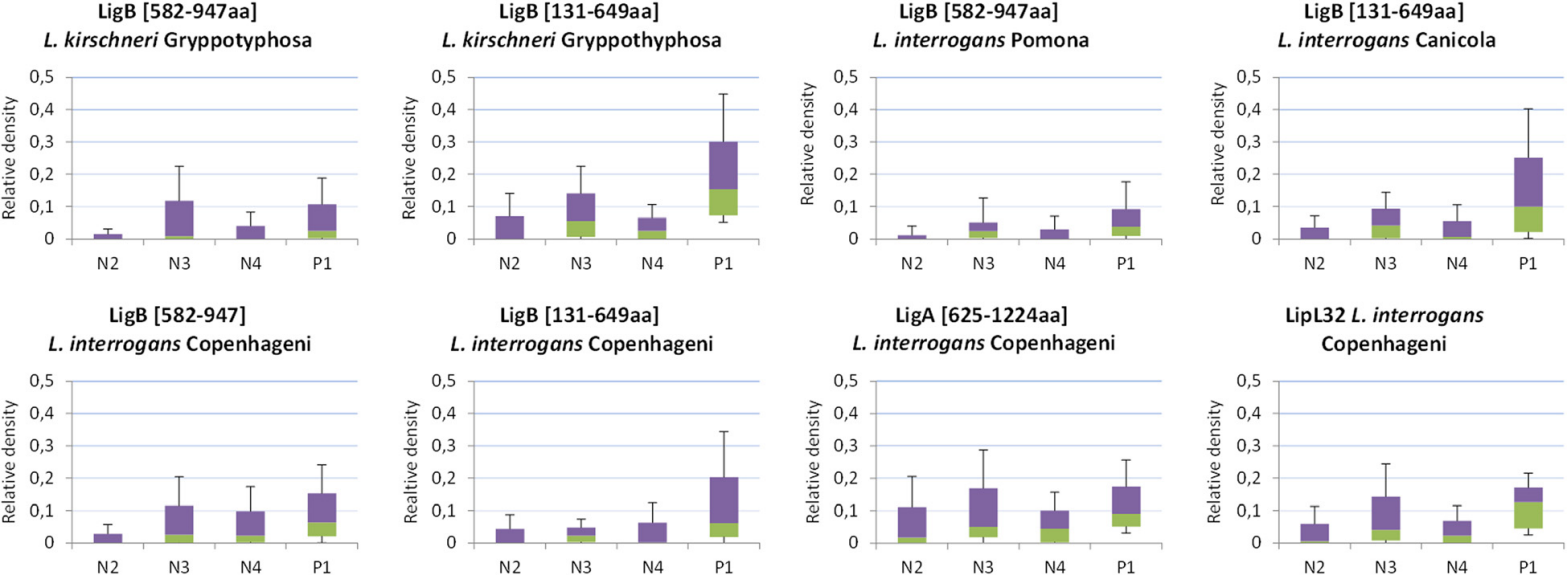
**Box plots representing the overall reactivity detected for each group of samples against the recombinant proteins.** Bars indicate the maximum or minimum values; the purple area indicates the 25^th^ percentile; the green area indicates de 75^th^ percentile; the line between purple and green areas indicates de median. Sample groups: P1, seropositive dogs; N2, housed vaccinated dogs; N3, stray dogs; N4, dogs with other febrile syndromes.

The purified recombinant proteins used for printing the MAPIA strips were analyzed by SDS PAGE 12.5% and showed high homogeneity (Figure 1). The MAPIA densitometry results showed that, in general, group N2 (housed vaccinated dogs) had lower IgM background against the recombinant proteins than groups N3 (stray dogs) and N4 (dogs with other febrile syndromes) and group P1 (seropositive dogs) had a stronger IgM reactivity (Figure 2). The reactivity detected for group P1 (seropositive dogs) against LigB [131-649aa] *L. kirschneri* Gryppotyphosa, LigB [131-649aa] *L. interrogans* Canicola and LipL32 *L. interrogans* Copenhageni were the most intense, with a higher median reactivity when compared to the control groups (Figure 2).

Using a cut-off of 1.5 standard deviations above the average reactivity of the control samples, we were able to establish sensitivity and specificity values for each of the recombinant proteins studied when comparing the seropositive group (P1) with each of the control groups (N2, N3 and N4). The results are summarized in Table 1. The specificity did not vary significantly when analyzing the different control groups. Conversely, sensitivity varied considerably, showing lower values when group N3 (stray dogs) was the considered group. Even though sensitivity is strictly related to the number of true positive samples, that is, reactivity among the reference samples in group P1, the use of different control groups lead to different cut-offs, which may ultimately influence the sensitivity. Indeed, background reactivity was higher in group N3 (Figure 2), increasing the cut-off value compared to groups N2 and N4 (Table 2). In general, Lig recombinant proteins [131-649aa] *L. interrogans* Canicola, Lig [131-649aa] *L. kirschneri* Gryppotyphosa and LipL 32 *L. interrogans* Copenhageni provided best sensitivity rates, for all control groups, except for group N3, in which Lig [131-649aa] *L. interrogans* Copenhageni overpassed LipL32.

**Table 1:** **Diagnostic performance of each antigen for all the control groups studied**

**Antigens**	**P1 vs N2**		**P1 vs N3**		**P1 vs N4**		**P1 vs N2, N3 and N4 combined**	
	**AUC**	**Se/Spe**	**AUC**	**Se/Spe**	**AUC**	**Se/Spe**	**AUC**	**Se/Spe**
LigB [582-947aa] *L. interrogans* Pomona	0.841	63/90	0.636	27/90	0.781	43/89	0.749	40/90
LigB [131-649aa] *L. interrogans* Canicola	0.879	73/87	0.763	53/93	0.840	70/89	0.826	67/91
LigB [582-947aa] *L. interrogans* Copenhageni	0.875	67/90	0.682	27/90	0.704	33/95	0.764	37/91
LigB [131-649aa] *L. interrogans* Copenhageni	0.827	63/87	0.707	47/87	0.811	57/89	0.777	50/87
LigA [625-1224aa] *L. interrogans* Copenhageni	0.875	63/93	0.654	20/90	0.714	40/95	0.764	37/89
LipL32 *L. interrogans* Copenhageni	0.951	77/93	0.787	43/93	0.869	70/95	0.869	60/91
LigB [582-947aa] *L. kirschneri* Gryppotyphosa	0.759	63/90	0.601	27/97	0.742	53/89	0.695	40/92
LigB [131-649aa] *L. kirschneri* Gryppotyphosa	0.919	80/93	0.787	57/93	0.861	70/89	0.855	63/90

Se: sensitivity; Spe: specificity. Sample groups: P1, seropositive dogs; N2, housed vaccinated dogs; N3, stray dogs; N4, dogs with other febrile syndromes.

**Table 2:** **Established cut-off for each of the proteins and control groups used in this study**

**Sera**	***L. interrogans***						***L. kirschneri***	
	**LigB [582-947aa] Pomona**	**LigB [131-649aa] Canicola**	**LigB [582-947aa] Copenhageni**	**LigB [131-649aa] Copenhageni**	**LigA [625-1224aa] Copenhageni**	**LipL32 Copenhageni**	**LigB [582-947aa] Gryppothyphosa**	**LigB [131-649aa] Gryppothyphosa**
*MAT-negative dog groups*								
N2	0.024	0.033	0.023	0.034	0.076	0.044	0.010	0.051
N3	0.079	0.087	0.101	0.072	0.180	0.127	0.072	0.134
N4	0.046	0.052	0.081	0.050	0.111	0.077	0.025	0.086
*MAT-negative dog groups combined*								
Groups N2, N3, N4	0.056	0.064	0.075	0.055	0.134	0.092	0.046	0.100

Sample groups: P1, seropositive dogs; N2, housed vaccinated dogs; N3, stray dogs; N4, dogs with other febrile syndromes.

**Figure 3:**
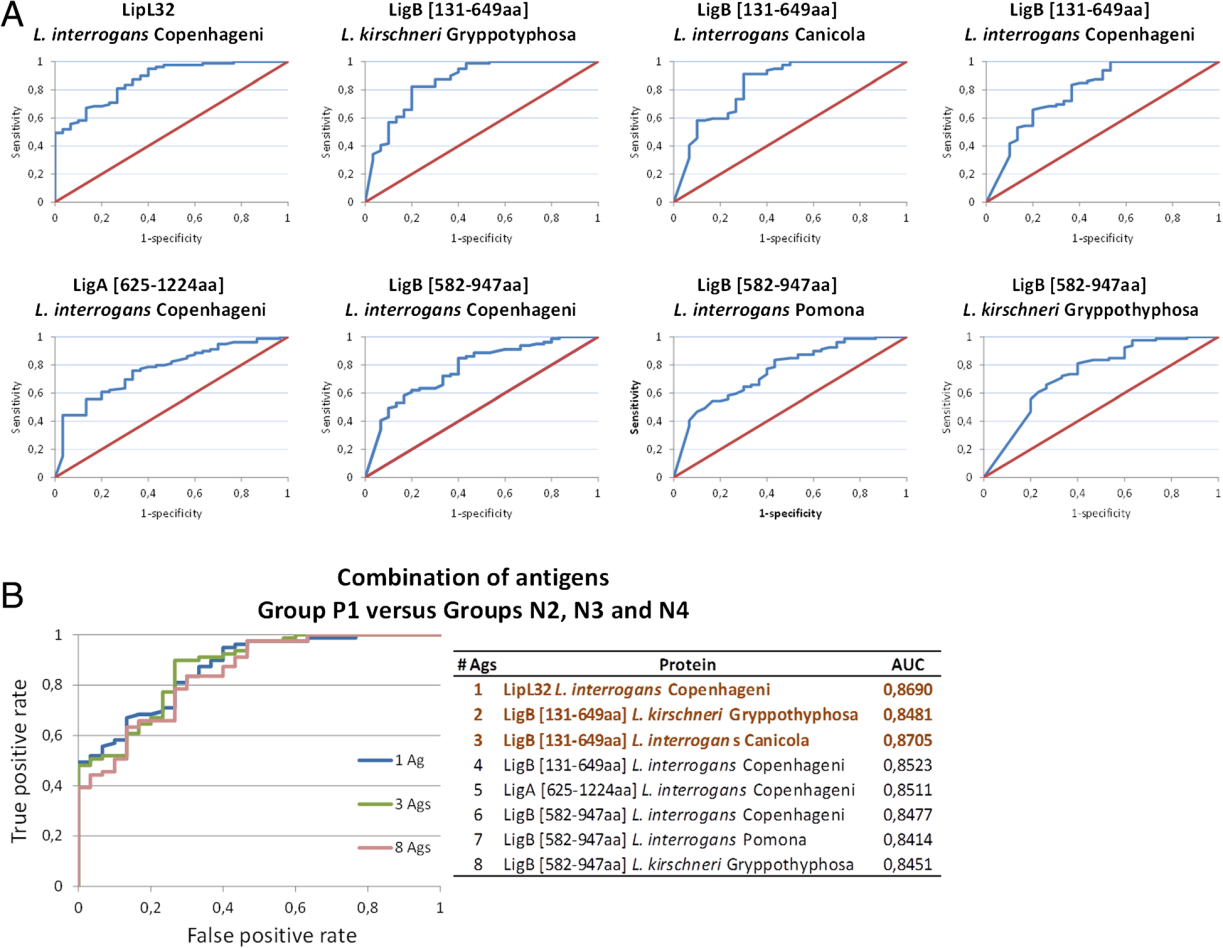
**ROC curves showing the diagnostic accuracy of each recombinant protein analyzed individually (A) or in combination (B) when combining the 3 control groups N2, N3 and N4 as a single group.** In **A**, proteins are sorted from left to right by decreasing AUC values. The combination of the first 3 antigens (**B**, dark brown) provides best accuracy. Sample groups: P1, seropositive dogs; N2, housed vaccinated dogs; N3, stray dogs; N4, dogs with other febrile syndromes.

**Figure 4:**
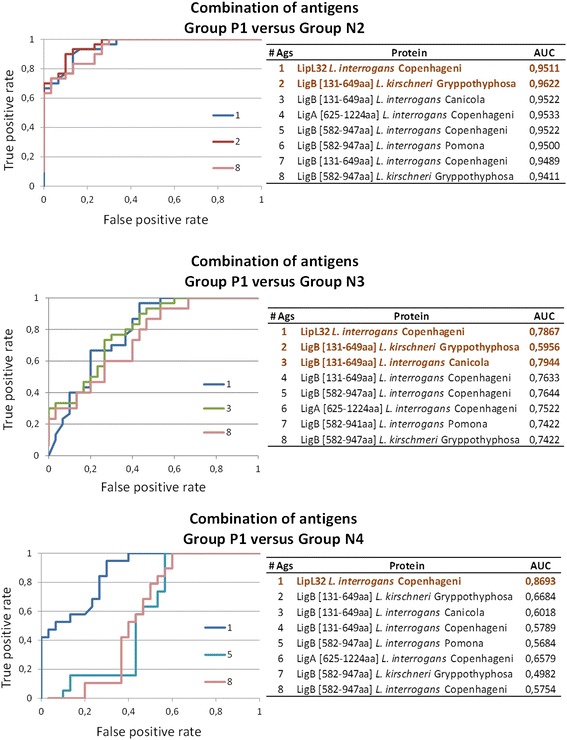
**ROC curves showing the diagnostic accuracy of the recombinant proteins when analyzed in combination.** Proteins were ranked by decreasing individual AUC and the impact of adding antigens, one at a time, was analyzed. The best combination is shown in dark brown. Sample groups: P1, seropositive dogs; N2, housed vaccinated dogs; N3, stray dogs; N4, dogs with other febrile syndromes.

In order to assess the diagnostic accuracy of the recombinant proteins, individual antigen ROC curves were generated and the corresponding AUC was determined. Antigens were then ranked by decreasing AUC and multiple antigens ROC curves were generated. Figure 3A shows the individual ROC curves for each of the recombinant proteins when we compared the seropositive dogs (group P1) against all the other groups combined as a single group (groups N2, N3 and N4). The AUC values are shown in Table 1. LipL32 *L. interrogans* Copenhageni provided best accuracy (AUC = 0.869), followed by LigB [131-649aa] *L. kirschneri* Gryppotyphosa (AUC = 0.855) and LigB [131-649aa] *L. interrogans* Canicola (AUC = 0.826). Furthermore, when group P1 was compared to groups N2, N3 and N4 separately, the same antigens showed best performance. The combination of those three antigens improved the diagnostic accuracy when considering all control samples in a single group (Figure 3B) and also when group P1 was compared to group N3 (stray dogs, Figure 4). Using group N2 as the control group, LipL32 and LigB [131-649aa] *L. kirschneri* Gryppotyphosa provided the best performance. When we consider group N4 (dogs with other febrile syndromes) as control, however, the addition of extra antigens did not impact the diagnostic performance of LipL32 (Figure 4).

## Discussion

In the present study we used three different groups of seronegative samples. Group N2 was a very well-defined group, with reliable information regarding shelter, immunization and veterinary assistance. Although Rio de Janeiro, as other tropical cities, is an endemic city for leptospirosis and it is not possible to exclude the possibility of previous contact of those dogs with leptospires, those animals present a low epidemiological risk of infection due to smaller chances of direct or indirect contact with rodents. Accordingly, higher sensitivity and specificity rates were obtained when the seropositive group P1 was compared against group N2. It is also interesting to highlight that all animals in group N2 had been vaccinated within 1 year before sample collection and, therefore, the antigens with high diagnostic performance identified when group N2 was used as control were actually able to differentiate between a current acute leptospirosis case and a vaccinated non-diseased dog.

Conversely, animals from Group N3 (stray dogs) presented the lower accuracy results. Information regarding the epidemiological status of those animals was very scarce, as well as data about previous diseases or immunizations history. Therefore, those dogs may have been exposed to many variables, and possible biases cannot be excluded. Nevertheless, it is important to note that in the daily practice information may not be available for the practitioner when a decision regarding the diagnostic test must be taken. In that situation, results of group N3 represent a non-ideal but very common scenario in veterinary practice, justifying its inclusion in the present study.

Noteworthy that in all comparative prospectuses LipL32 presented good results and discriminatory abilities. Although its potential for diagnosis tests is still controversial, the protein is known to be an immunodominant antigen in leptospirosis, particularly for the acute syndrome of the disease in human beings [12,15-17]. It has been reported that IgM antibodies specific to the C-terminal region of LipL32 can be detected during the acute infection, so that LipL32 could be effectively used as a diagnostic marker [18]. Furthermore, it has been successfully employed as an amplification target in molecular diagnosis (PCR) in human and animal samples, with excellent specificity, since it is present exclusively in pathogenic leptospires [19,20].

LipL32 alone or associated to LigB [131-649aa] of *L. interrogans* Canicola and/or *L. kirschneri* Gryppotyphosa showed the best discriminatory potential. The fragment LigB [131-649aa] of *L. interrogans* Canicola and/or *L. kirschneri* Gryppotyphosa is encoded by a genetic region (131-649aa) that shares 100% identity with LigA. Though LigA is present only in *L. interrogans* and *L. kirschneri*, LigB has been detected in all pathogenic *Leptospira* species [11]. Furthermore, the fragment LigB [131-649aa] has been described as a highly sensitive marker for leptospirosis in humans, particularly in the first week of the course of infection [8].

Our findings for canine leptospirosis are in contrast with recent studies conducted in human beings, in which the LigB [582-947aa] and LigA [625–1224] proteins were the basis of a novel rapid test and showed promising results [9]. Here, we show that those fragments are not as strongly antigenic in dogs.

The predominance of seroreactivity against serogroup icterohaemorrhagiae detected by MAT was expected and agrees with previous studies conducted with clinically suspect Brazilian dogs [21]. As a consequence, even though it was not the original purpose of this study, the MAPIA analysis performed here took into account the incidental syndrome of canine leptospirosis. Therefore, it is important to highlight that our results may not represent the chronic/subclinical disease that is determined by the host-adapted serovar Canicola. It is interesting, though, the fact that LigB [131-649aa] of L*. kirschneri* Gryppotyphosa and *L. interrogans* Canicola presented better results than LigB [131-649aa] *L. interrogans* Copenhageni. Although theoretically a protein obtained from Copenhageni strains should be more useful for detecting dogs infected by icterohaemorraghiae serogroup, it was not observed in the current study. Noteworthy that LigB obtained from *L. kirschneri* has a 91% identity with respect to *L. interrogans* serovar Copenhageni [11], and the region between amino acids 131-649aa of LigB fragment corresponding to LigB domains 2–6 has an even higher identity (96%), which could possibly explain the obtained results.

## Conclusions

MAPIA identified important immunodominant antigens in canine leptospirosis. LipL32 presented the most consistent results in all analysis, and may represent a major candidate for diagnostic tests. Alternatively, LigB [131-649aa] of *L. interrogans* Canicola and *L. kirschneri* Gryppotyphosa may increase accuracy when present in a combined antigenic formula for detecting leptospirosis in dogs.

## Methods

### Study design

Canine serum samples were collected in veterinary hospitals in Rio de Janeiro, Brazil. After collection, MAT was conducted as described later in this section in order to determine dog’s sero-reactivity to leptospires. Samples were grouped according to patient’s data provided by the assistant veterinarian (when available) regarding anamnesis, clinical presentation and vaccination history; and on the MAT results. Finally, the Multi-antigen Print Immunoassay (MAPIA) was performed and the samples’ reactivity to different recombinant proteins of the pathogenic strains *Leptospira interrogans* serovar Copenhageni, Pomona and Canicola and *Leptospira kirschneri* serovar Gryppotyphosa was evaluated.

### Ethics statement

The study protocol was approved by the Ethics Committee on Animal Use at Fluminense Federal University, Brazil (Protocol 154/2011). Written or verbal consent from the owners of the dogs was received to use blood in this study.

### Animal

Blood samples were obtained from 109 adults 2-17y, including (i) 30 sero-reactive dogs with MAT titer ≥800, showing clinical signs compatible with anorexia, fever, dehydration and icterus (based on the clinician’s judgment) and which may or may not have been vaccinated (Group P1); (ii) 30 housed non-diseased dogs with titer ≤100, which had been vaccinated (commercial bacterins) in the last year and showed no clinical alteration, so considering this group as healthy vaccinated animals (Group N2); (iii) 30 stray dogs with MAT titer ≤100 and unknown history of vaccination, considering this group as unvaccinated healthy animals (Group N3), and (iv) 19 dogs sero-negative for leptospirosis (MAT titer ≤100) but presenting a different febrile syndrome (Group N4), as canine distemper disease (n = 1), ehrlichiosis (n = 8), anaplasmosis (n = 6) or babesiosis (n = 4). The febrile syndromes were diagnosed by reference laboratories through the direct detection of the pathogenic agent on blood and/or commercial ELISA serological tests. Animals’ informations are summarized in Table 3. The diseases have similar clinical alterations to leptospirosis, justifying the importance of this group in the study.

**Table 3:** **Groups of canine serum samples used in this study**

**Group**	**# serum samples**	**Diagnosis**	**MAT titer**	**Housing**	**Age range (years)**	**# vaccinated dogs in the last year**
P1	30	Leptospirosis	≥800	N/A	2-17	4
N2	30	Healthy	≤100	Yes	2-16	30
N3	30	Healthy	≤100	No	N/A	0
N4	19	Other febrile syndromes	≤100	N/A	2-15	18

N/A: Not available.

The blood was withdrawn from cephalic vein. Serum was then separated by centrifugation and stored at −20°C until use.

### Microscopic agglutination test

For detection of anti-*Leptospira* antibodies, microscopic agglutination test (MAT) was conducted according to the recommendations for international standards [22]. The complete panel included 28 serovars representing all the 24 known serogroups (from Royal Tropical Institute - KIT, Amsterdam, Netherlands). Bacteria were cultured in liquid Ellinghausen-McCullough-Johnson-Harris (EMJH) and used as antigens. Each serum sample was initially diluted 1:50 in buffered saline and 50 uL of this solution were transferred into vinyl microplates containing 96 wells (Corning Incorporated, Corning, NY, USA). Immediately after, an equal volume of each antigen was added to the corresponding well, with a final dilution of 1:100. The microplates were incubated at 37°C for 90 minutes and observed under dark-field microscopy. Serum samples were initially analyzed at a dilution of 1:100, and those that showed agglutination level equal to or higher than 50% were then diluted again in a geometrical ratio of two (1:200, 1:400 and 1:800). Samples were considered as reactive when reached titers of 200 or 400. Reactions that presented titres ≥800 were considered as strongly reactive and considered as an indication of acute infection [22].

### Cloning, expression and purification of recombinant proteins

DNA fragments obtained by PCR amplification were cloned into the pET100-TOPO plasmid (Invitrogen) for the expression of recombinant proteins containing an N-terminal His_6_ tag. All plasmid constructs were confirmed by DNA sequencing using an ABI 3100 sequencer (Applied Biosystems). The Lig plasmid constructs were cultured at 37°C to the mid-log phase, and expression was induced using isopropyl-β-D-thiogalactopyranoside (IPTG) at a final concentration of 1 mM. The cultures were then grown at 28–30°C, depending on the protein. Cells were harvested by centrifugation at 10,000xg and resuspended in 20 mM Tris, 1 mM EDTA pH 8.0 and 0.1% Triton X-100. After incubating for 30 minutes at 4°C, the cells were disrupted by sonication (Sonics & Material). The lysate was centrifuged at 10,000 × *g* for 20 minutes at 4°C. The recovered soluble fraction was applied to a Ni^2+^-charged column (Histrap HP, GE Healthcare) in a High pressure liquid chromatography (HPLC) system and washed with 10 column volumes of buffer (PBS, pH 7.4, 20 mM imidazole). The following stages had been made using steps varying from to 20 mM to 300 mM stepwise gradient of imidazole until the protein was eluted. The purified proteins were checked for homogeneity in 12.5% SDS-PAGE and quantified by the BCA method (BCA Thermo Scientific Pierce, Rockford, IL, USA) according to the manufacturer’s recommendations.

### Recombinant proteins

Eight recombinant proteins, designed based on the domains of the Lig proteins, were employed in this study: LigB [582-947aa] from *L. interrogans* serovar Pomona, *L. interrogans* serovar Copenhageni and *L. kirschneri* serovar Gryppotyphosa, LigB [131-649aa] from *L. interrogans* serovar Copenhageni, *L. interrogans* serovar Canicola and *L. kirschneri* serovar Gryppotyphosa, LigA [625-1224aa] from *L. interrogans* serovar Copenhageni and LipL32 [1-272aa] from *L. interrogans* serovar Copenhageni. These fragments were cloned, expressed in *Escherichia coli* expression system and purified by affinity chromatography as described above.

### Multi-antigen print immunoassay

The assay was performed as described previously [23]. Briefly, antigens were immobilized on a nitrocellulose membrane (Schleicher & Schuell, Keene, N.H.) so that each strip contained 100 ng of each of the proteins. Phosphate buffer saline was spotted on the nitrocellulose membrane as a negative control reaction (blank). A semi-automatic micro-aerolization device (Automatic TLC Sampler 4, CAMAG, Muttenz, Switzerland) was used to generate parallel bands with no visible marks. The membrane was cut into 0.4 cm wide strips perpendicularly to the antigen bands. Protein A was added as a control band of antigen-antibody reaction. The strips were blocked overnight with 4% reduced-fat bovine milk diluted in 0.05% PBS Tween 20 (Sigma-Aldrich) and then incubated for 2 h at room temperature with individual serum samples (diluted 1:400 in blocking solution) and 150 ug/ml of *E. coli* extract. After washing, the strips were incubated for 1 h with alkaline phosphatase–labeled anti-dog IgM antibody (Alkaline Phosphatase Dog Conjugated Antibody IgM, Bethyl Laboratories, TX, USA) diluted 1:50,000 in 0.25% BSA-0.05% PBS Tween 20 (Sigma-Aldrich). After washing, the strips were incubated with a substrate for alkaline phosphatase (Western Blue Stabilized Substrate for Alkaline Phosphatase, Promega, WI, USA) for 10–15 minutes and the reaction was stopped with distilled water. The reactivity was quantified by densitometry (Bio-Rad GS-800 Calibrated Densitometer, Hercules, CA, USA) and data analysis was performed with Quantity One 1-D Analysis software, Bio-Rad.

### Statistical analysis

For densitometry analysis, relative density was determined by subtracting the background detected for the blank reactions from the reactivity against the recombinant proteins. Cut-off points were established considering the average reactivity of the control samples plus 1.5 standard deviations. Sensitivity and specificity were calculated and defined for each control group individually as well as in combination. Receiver operating characteristic (ROC) curves were made with the “ROCR” R package in the R environment using the “leave one out cross-validation” (LOOCV) approach. ROC graphs were created by plotting the true positive rate (sensitivity) against the false positive rate (specificity) at various threshold settings in order to illustrate the recombinant proteins diagnostic performance, which was assessed by the area under the curve (AUC).
